# Discharged. Not Where to Lay His Head

**Published:** 1886-10-23

**Authors:** George Fleming


### DISCHARGED.

### "NOT WHERE TO LAY HIS HEAD."

By George Fleming.

V.

The old ex-Chartist looked hard at Lemon for a minute or too, without recognising him. Then he took his spectacles off and laid them down on the bench before him and nodded. It was one of his peculiarities never to use glasses for his work; he only put them on to examine the faces of the people who spoke to him.

"Thought you had gone away. Some chap here said he had seen you in America," he observed drily.

This grey-headed old fellow had a story, too, of his own; but he was old. The greater part of the people who met him had either never heard of him, or had long ago forgotten his name. The younger men in the shop used still to draw him out about Red Republicanism, at times, and the schemes of '48; but to his employers he was simply one of the steadier lot of workmen, and, in his way, a skilful carver. He went on now, all the time Lemon was speaking, cutting away steadily at the corner of a monstrous frame.

"And so," he said, "you thought you'd find Mr. Thomas and the job all waiting for you whenever you chose to come in for them, eh?"

The last of the gilders had struck work, and lounged or hurried out of the place as his fancy led him, while Lemon was still explaining his position. He looked all about him now, and the empty tables, the sunlight shining through the dusty window panes upon the tools left lying where they had been thrown down; everything he looked at seemed to add its touch of definiteness to his disappointment.

He sat down upon the nearest bench, thrust his hands into his empty pockets. "Well! a man can't do more 'n ask for work," he said sulkily.

"Think not? In my time we used to make it for ourselves. We didn't expect to see roast chicken provided by Act of Parliament." He paused to measure an angle very carefully. Then he looked up, rubbing the back of his hand across his shaggy eyebrows, and a good broad smile tempered all the severity of his wrinkled, melancholy old face.

"There, lad; don't be huffish. Wait a bit. You and me was never particular pals that I know of, but I was used to seein' you goin' in an out o' here pretty often, and you have a kind o' look about you that puts me in mind of some one I used to know. Now, look here"—he put on his spectacles —"Have you got any money?"

"Not a ha'penny," returned Lemon, looking him straight in the eyes.

"Well, well. That's a badish look-out. Mr. Thomas ain't often got work to give away on a sudden. And this lady as you was tellin' me of. You've posted that letter?"

"Not yet. I'll get the address this afternoon."

"See there, now! Ah, but the thing with swells is to treat them accordingly. I've known some of them,—spoken to 'em, I mean,—and, Lord bless you, lad, there was no more end to their kindness than there is to that blessed sunshine. I'm not sayin' they're all like that, an' good feelin'—any more than we are. But there's some. And any man who says 'no' to that, you can tell him it ain't true. But they're ignorant o' many things. And they're different from us; it stands to reason. Now, this lady of yours: she may answer you in a week, if she is kind, and not too busy; an' if you wait for that job with empty pockets, why, where are you?"

Where, indeed? Lemon sat there kicking the shavings about with his feet in silence.

"Mr. Thomas, he likes giving a man a job when he takes it quiet like, as if it didn't matter; not when he snaps at it like a starving dog. He's just, but he won't pay a bigger price for a man than the man asks for himself. If he knows he can have a thing when he wants it, he waits *till* he wants it; an' that's the way with the most of 'em. Lord! I've known such a lot of people wouldn't help a hungry man o' Monday because they weren't sure they could do it all the days of the week; they thought as long as they never helped one at all there wasn't any responsibility."

He looked critically at Lemon's flushed cheeks and bright, glassy eyes. "There's a bit of gilding to be done on them chair backs; they'll be ready for it Toosday. It's a matter of fifteen shillins the job. They give it to me to take home, but—— Mind, it's due here on Saturday night, my lad; sharp, and no skulking. Mr. Thomas knows I never break my word."

Lemon looked up. "Do you mean to say"—he began, in a breathless sort of voice.

"Ay, you can take 'em. You can get the gold leaf Monday night an' be ready to begin.—Some folks would call me an old fool for my pains, but I tell you what it is, my lad, if a matter of three half-crowns would help you and keep you goin', why, I'll advance it on the work myself.—You see if I don't!" the old man called out fiercely.

They walked a bit of the way down Oxford Street together. Lemon was going back to Exhibition Road for Miss Thornhill's address. As they were about to part old Dick checked him for a moment. "Look a here!" he said roughly, "the cut of your face puts me in mind o' some one you never heard of. That don't seem much of a reason for lookin' after you; does it now? But I took a fancy to you, years ago, all the same. And I'll give you one piece of advice. I heard you tellin' those chaps you'd taken the pledge. Well, then,—keep it!"

Lemon laughed.

"Why, I'm in a coffee tavern now," he said gaily.

The ex-Chartist shook his grizzled head. "Lad, to-morrow's Sunday. They call it the Lord's Day in the churches, and maybe it is so,—there. But out in the London streets it's a day that's been made for the devil. Nothing to do; no place to go to; an' at every street corner a swingin' door opening straight down to hell."

He stood still, with his eyes fixed on the pavement. When Lemon had already moved off a few paces he called him back. "Hi! look here." Lemon returned, wondering.

"It's another 'arf-crown. That makes four. It ain't so much, an' it's all I can do for you, an' I wish to God they hadn't discharged you at the very end of the week," he said, staring at Lemon with an odd, embarrassed tenderness.

That afternoon Lemon got Miss Thornhill's address. Then he lingered on the doorstep for another ten minutes talking to Mary Parker. She had taken off her cap, and tied a bit of purple ribbon in her hair; she was prettier even than he remembered her. She called him "Mr. Lemon" now, when she opened the door to him. He told her all about his lucky encounter with Chartist Dick, and she was not at all astonished to hear about the money; only she wanted to know why the men called him Old Five Points, and when Lemon had explained it to her, "Lor! I suppose you know a lot of things?" she said, looking up at him shyly through her dark eyelashes.

Lemon laughed in a superior fashion. "Oh, I daresay I've read a good bit in my time. We have to know some things in our trade; for restorations and such."

"Ah, they'll look up to you a good deal, I'll be bound! They must have been glad to get you back," she said, simply; and he laughed and reddened with pleasure. Her admiration was sweet to him, like wine.

She could not linger in talk, for her aunt was in the house, she said, and would want to know what was keeping her. But when he left her he did not go home, but into Kensington Gardens. There were very few people walking about there, although the sunshine rested upon the grass as warmly as in summer. Lemon threw himself down on the ground under a tree, and the little dog lay close beside him. Overhead the great strong branches crossed and crossed against the pale, sunny blue of the sky. Now and then a yellow leaf detached itself mysteriously, noiselessly, from the stem which held it, and floated down, resting where it fell—upon his hand, or on the sheep-nibbled turf beside him. Some children were playing together, not far off, near the Round Pond. Their voices sounded very pleasant. The very noise of the High Street was mellowed, it fell subdued upon the ear to the murmur of a hive of bees.

He began thinking of his dead wife as he lay there, smoking. It seemed such a long, long time since first they met. The children he had never cared about very much; they had always been ailing; and when he thought of his wife it was not as their mother that he remembered her, but as the girl he had fallen in love with—with little delicate ways almost like a lady. And then he began comparing her with Mary. She was delicate too, but that was his taste; he had always liked women's ways to be soft and pleasant to look at. "None of your music-hall style for me," he thought, and laughed idly. The clear, clear blue of the sky overhead seemed like an assurance of peace and protection. Something of that same sense of fellowship with all men which had moved him so strongly yesterday—a relief from physical pain which transformed the commonest incident into an act of personal kindness—returned to him as he lay thus. He threw his arm across the warm, smooth back of the little dog. "So you wouldn't leave me, you little rascal," he said aloud; "you knew when you were well off, eh?" He remembered the young gentleman who had given him tobacco in the train yesterday; the landlady who had trusted him; and old Dick, who had lent him the money. He looked straight into the dog's liquid brown eyes, and a vague feeling of the unity of all created life, the likeness between them both, unimportant waifs in a preoccupied universe, loving, irresponsible recipients of chance kindness, made him turn and stroke the quivering little creature near him with a renewed affection. "Poor little chap. Poor dog," he said, gently, and when Spot licked his hand it was as if they understood one another.

After a time he fell asleep. It was dusk when he awoke. The great branches rustled overhead; at first he could not remember where he was. It was a very dark autumn night. The air was moist, and the rising wind made the lines of gas-lamps flicker. He strolled back across the damp grass, by the edge of the Serpentine, and so on into Knightsbridge, not thinking of anything in particular. But, near the gate, a small crowd was gathered about a fallen dray. Both horses were down, kicking and struggling in the darkness; the driver was standing by their heads, and a couple of policemen mounted guard over a huge cask which had had a hole knocked in it. Two or three men in their shirt-sleeves were dipping out its contents with pails, and the whole street reeked with the heavy smell of beer. Lemon stood with the others, looking on, and he laughed with the rest, when a voice cried out, "By Saint Patrick! but if they would only use me instead of them buckets!" He no longer disliked the smell; the unpleasant impression of the morning had entirely passed away.

That evening dragged. It was a pleasure in its way to ask the be-ringleted and disdainful barmaid for change for his half-sovereign. But, after he had paid her, he wished that he had kept the money. The people who dropped into the coffee-room were, for the most part, regular customers; a good many of them were elderly women with boys. There was very little talking. A dreadful dulness, a respectable deadly depression was in the very air they breathed; it seemed to hang about the folds of their decent dresses; it even affected the boys.

Lemon went very early to bed. For a long time he could not sleep; there was storm in the air, and a feeling of thunder. Towards morning his chest began aching dully; and then, all of a sudden, as he lay tossing about on his narrow bed in the hot, stuffy room, there flashed before his eyes a tantalising vision of the drink which had been offered him in the morning. He could see its crested foam, and smell the cool, bitter fragrance. After a time, he could not stand it any longer; he got up and groped about in the dark for the jug of water. It was lukewarm to the taste; but after he had bathed his face and hands in it, the physical sensation of actually *seeing* the beer, there, before his eyes, gradually left him,—melting away into the dreams of a feverish sleep. But each time that he woke in the night the same vision returned with varying vividness.

*(To be continued.)*

